# Muriate of Cocaine

**Published:** 1885-02

**Authors:** E. G. Betty


### THE DENTAL REGISTER.

VOL. XXXIX.] FEBRUARY, 1885. [No. 2.

### COMMUNICATIONS.

### Muriate of Cocaine.

BY E. G. BETTY, D.D.S.

Rob't Sattler, M. D., of Cincinnati, has been conducting a series of experiments with this new anæsthetic at his clinics. His results were published in the Cincinnati *Lancet and Clinic*, for November 1st, of last year. A five per cent solution produced these results:

1. The instillation is followed, in every case, by more or less smarting and burning, which soon gives way to a feeling of coolness.

2. In from three to twenty minutes impairment of the sensibility of the cornea, followed by a period, varying in duration, during which all reflex irritability is lost or temporarily abolished, and anæsthesia of the anterior superficial structures of the eye appears complete. This period varies, and rarely exceeds one hour; the average duration among ten cases was twenty-five minutes.

3. During this period all reflex irritability is lost; the cornea can be touched with unyielding substances, wood, paper, the point of a knife, and active, spasmodic movements of the eyelids or profuse lachrymation, the almost invariable concomitants of reflex action, are not observed.

5. A peculiar pallor of the conjunctiva, and whiteness of the sclera, together with contraction or diminished prominence of the blood vessels of the conjunctiva.

Dr. Sattler then details a number of cases in which the anæsthetic was used in the eyes, giving minute details as to length of time required to produce anæsthesia, duration of same, character of operation performed, etc.

What a glorious thing it would be for dentistry, if equally satisfactory results could be obtained in obtunding the sensibility of living dentine during the preparation of cavities for filling! Though we have not, as yet, made any experiments (which we will shortly), we are inclined to believe that for our purposes, muriate of cocaine will become the rage for a while and then be relegated to the long list of abandoned pain obtunders. This conclusion seems somewhat hasty and apparently without foundation; but if the reader will stop to think for a moment he will see that it is upon *vascular* tissues that the muriate has produced these wonderful effects. Read again the fifth section of Dr. Sattler's paper, and note that the anæsthetic produces a marked *anœmia* of the tissues, and which, no doubt, gives rise to the sense of coolness that succeeds the smarting and burning immediately after the instillation. Any tissue deprived of its blood, loses its functions and sensibility; a profuse hemorrhage produces unconsciousness and insensibility to outside influences. If, then, as this hasty examination seems to indicate, vascularity is an essential, then muriate of cocaine can not act as an anæsthetic upon dentine, though it is a true anæsthetic and not an anodyne merely.

Dr. Will. Taft flooded a cavity with a ten per cent solution over an exposed pulp, allowing the parts to remain in that condition for half an hour, during, and at the end of which time, neither pulp nor dentine showed the slightest diminution of sensibility.

It may be that in this case the foramen at the apex was so constricted that the muriate could not produce anæmia of the pulp, and in consequence, no anæsthesia. In other instances, no doubt, it will anæsthetize the pulp, and permit its extirpation without pain.

There are other uses to which the dentist may apply the muriate of cocaine with satisfactory results, as: for extraction, by deadening the sensibility of the surrounding gums; drilling into the the antrum, or trephining an abscess. These can be, and no doubt will be, tested by experiment. It has been found that anæsthesia of a part may be produced by hypodermic injection of the cocaine near the nerve supplying the part to be operated upon, as Dr. Hepburn relates in his experiments, published in the January number of the *Independent Practitioner.*

Topical application of the agent to mucous surfaces gives astonishing results, and it is safe to say that for many purposes muriate of cocaine has, in the short space of two or three months, earned for itself a prominent and permanent place in our Materia Medica, even should experience show that in our haste and enthusiasm we may have over-rated its powers.

